# The experience of social exclusion in women with a history of suicidal acts: a neuroimaging study

**DOI:** 10.1038/s41598-017-00211-x

**Published:** 2017-03-07

**Authors:** Emilie Olié, Fabrice Jollant, Jeremy Deverdun, Nicolas Menjot de Champfleur, Fabienne Cyprien, Emmanuelle Le Bars, Thibaut Mura, Alain Bonafé, Philippe Courtet

**Affiliations:** 1 Department of Emergency Psychiatry & Post Acute Care, Academic hospital of Montpellier, Montpellier University, & INSERM U1061, Montpellier, France; 2 Department of Psychiatry, Academic hospital of Nimes, Nimes, France; 3 McGill University, Department of psychiatry, and Douglas Mental Health University Institute, McGill Group for Suicide Studies, Montreal, Québec Canada; 4 Department of Neuroradiology, Academic hospital of Montpellier & U1051, Institut of Neurosciences of Montpellier, Montpellier, France; 5 I2FH & CNRS UMR 5221, University of Montpellier, Montpellier, France; 6 Neurosurgery, Academic Hospital of Montpellier & INSERM U1061, Montpellier, France; 7 Center for clinical investigation, Academic hospital of Montpelier, Montpellier University, Montpellier, France

## Abstract

Suicidal behaviors result from a complex interaction between social stressors and individual vulnerability. However, little is known of the specific neural network supporting the sensitivity to social stressors in patients at risk of suicidal acts. Using functional Magnetic Resonance Imaging, we investigated brain processing of social rejection in suicide attempters. Thirty-six euthymic women with a history of depression and suicidal behavior were compared to 41 euthymic women with a history of depression but no suicidal attempt, and 28 healthy controls. The Cyberball Game was used as a validated social exclusion paradigm. Relative to healthy controls, both patient groups reported higher levels of social distress related to the task, without significant differences according to suicidal status. Compared to patients without any history of suicide attempt and healthy controls, suicide attempters showed decreased contrast in the left insula and supramarginal gyrus during the exclusion vs. inclusion condition, after controlling for number of depressive episodes, medication, mood disorder type or social phobia. Our study highlights impaired brain response to social exclusion in euthymic female suicide attempters in regions previously implicated in pain tolerance and social cognition. These findings suggest sustained brain dysfunctions related to social perception in suicide attempters.

## Introduction

Humans have a fundamental need for social belonging that, when thwarted, has consequences on mental well-being^1–3^. Low social integration has been reported as a potential risk factor for suicide^4^, raising the question of the need to focus on an “outside- in” view to capture the dynamic influence between biological and environmental factors on suicidal behavior^5^. The majority of suicide victims had experienced at least one or more adverse life events within the last few months before their death^6^. Interpersonal conflicts, relationship breakdown and job loss or difficulties are among the most prevalent events, all social stressors confronting the individual with some form of social exclusion and a threat toward their social status. However, while these events are rather common in general, only the most vulnerable individuals are at increased risk of committing suicide when facing such stressors, suggesting that the way these individuals process social perception and interactions is key to understanding their fatal act. It has been suggested that all these psychosocial stressors trigger a high level of psychological pain, a frequent theme in suicide notes^7^, which, in turn, increases the risk of suicidal ideas and act^8^. Finally, interpersonal difficulties and social exclusion facilitates risky decision-making^9, 10^, a putative endophenotype of suicide^11^. For example, excluded individuals are more prone to eat non-nutritive foods and to avoid less tasty but nutritive foods that should be preferred for survival^12^. The study of social cognitions - including social rejection and associated psychological pain - and their neural basis is therefore crucial for understanding the suicidal vulnerability, which will shed light on potential therapeutic targets.

The vulnerability to suicidal behaviour has been associated with the dysfunction of several brain regions and cognitive processes. Notably, suicide attempters showed increased activation of the lateral orbitofrontal cortex following exposure to angry faces^13^, suggesting an over-evaluation of emotional negative cues; increased activity in the middle prefrontal cortex and anterior cingulate cortex when recalling the psychological pain experienced during the suicidal episode^14^; and reduced activation of the ventral prefrontal cortex during risky choices^15^ and the anticipation of rewards^16^, both associated with risky decision-making. A study in depressed patients suggests that psychological pain is associated with increased perfusion in the dorsolateral prefrontal cortex and in inferior frontal gyrus, but also in occipital cortex and in inferior temporal gyrus^17^. Altogether, it is hypothesised that the development of unbearable psychological pain following perception of social threat or rejection may lead to choose options (i.e. suicidal act) with short-term reward (i.e. relief from pain) in spite of the risks (i.e. death) in vulnerable individuals, partially relying on prefrontal cortex. Moreover, several neuroimaging studies in suicide attempters suggest that brain alterations associated with the suicidal vulnerability extend beyond the prefrontal cortex, notably the temporal and parietal cortices^18^, known to be involved in processing social cognitions^19^. However, to date, no study has specifically investigated response to social rejection in vulnerable individuals i.e. those with a history of suicidal acts.

In the present study, we used a validated paradigm of social exclusion, the Cyberball Game^20^. During this task, the participant plays a virtual ball-tossing game with two supposedly real other participants. However, he/she is not informed that he/she will progressively be excluded from the game by the two other participants who will continue to play together. A recent meta-analysis reported that three main areas were reliably recruited during the exclusion phase of the Cyberball task: the anterior insula, the anterior cingulate cortex, and the inferior orbitofrontal cortex^21^. Intriguingly, these results therefore show a relative overlap with the brain regions previously associated with the vulnerability to suicidal acts^22^. In order to focus on the vulnerability to suicidal acts, we compared patients with vs. without a history of suicidal acts to control for the effect of comorbid disorders (mood disorders here). Moreover, patients were euthymic at time of scanner to exclude the effect of acute depressive state and a large sample of participants was recruited to allow sufficient statistical power.

## Results

### Sociodemographic and clinical data

There were no between-group differences for sociodemographic variables (Table 1).

**Table 1:** Between-group comparisons for sociodemographic and clinical variables.

	Healthy Controls (n = 28)	Patient Controls (n = 41)	Suicide Attempters (n = 36)	Analyses		
	Median (min-max)	*Median* (*min*-*max*)	*Median* (*min*-*max*)	*KW*/*MW*	*p*	*Post hoc*
Age (years)	38.9 (22.6–50.9)	37.6 (19.7–50.7)	39.48 (19.4–54.2)	1.29	0.52	—
NART	22 (0–29)	22 (14–27)	22 (14–29)	0.37	0.83	—
Education (years)	15 (9–18)	15 (12–18)	14 (9–17)	1.93	0.38	—
HDRS	2 (0–20)	2 (0–26)	3.5 (0–18)	1.86	0.39	—
YMRS	0 (0–0)	0 (0–4)	0 (0–4)	2.52	0.28	—
BDI	0 (0–4)	3 (0–21)	3.5 (0–15)	27.18	<10^−2^	HC < PC, SA
BIS (total score)	42 (20–62)	45 (25–76)	49 (25–87)	4.07	0.13	—
STAI trait	29.5 (20–41)	45 (22–69)	47.5 (28–62)	36.79	<10^−2^	HC < PC, SA
STAI state	25 (20–40)	33 (22–75)	31 (20–45)	7.05	0.03	HC < PC,SA
STAXI trait	14.5 (10–21)	19 (12–28)	18 (11–32)	17.4	<10^−2^	HC < PC, SA
STAXI state	11 (10–30)	11 (10–28)	11 (10–30)	2.68	0.26	—
CTQ emotional neglect	6.5 (5–25)	11 (5–23)	15 (5–24)	19.98	<10^−2^	HC < PC, SA
CTQ emotional abuse	5 (5–22)	11 (5–25)	11.5 (5–22)	23.22	<10^−2^	HC < PC, SA
CTQ physical neglect	5 (5–11)	6 (5–16)	7 (5–23)	11.94	<10^−2^	HC < PC, SA
CTQ physical abuse	5 (5–13)	5 (5–16)	6 (5–23)	11.28	<10^−2^	HC < PC, SA
CTQ sexual abuse	5 (5–9)	5 (5–17)	5 (5–25)	3.9	0.14	—
Social distress (NTS)	54 (31–87)	65 (40–96)	63.5 (41–96)	8.7	0.01	HC < PC, SA
Number of depressive episodes	—	2 (1–50)	3.5 (1–13)	437	<10^−2^	PC < SA
Number of (hypo)manic episodes	—	0 (0–50)	1 (0–20)	602	0.27	—
Age of onset RRRS (most severe suicidal act)	—	24 (7–41)	20 (6–39)	573	0.19	—
- Risk subscore	—	—	9 (6–15)	—	—	—
- Rescue subscore	—	—	12 (10–14)	—	—	—
SIS (most severe suicide attempt)	—	—	15 (8–20)	—	—	—
RRRS (last suicidal act)						
-Risk subscore	—	—	7 (5–13)	—	—	—
- Rescue subscore	—	—	12 (9–15)	—	—	—
SIS (last suicidal act)	—	—	12 (5–26)	—	—	—
		% (*n*)	% (*n*)	*Chi2*	*p*	*Post hoc*
Bipolar disorder	—	36 (15)	56 (20)	*2.8*	0.07	—
Current panic disorder	—	4.8 (2)	11.2 (4)	NA	—	—
Agoraphobia	—	7.2 (3)	11.2 (4)	NA	—	—
Current social phobia	—	4.8 (2)	16.8 (6)	NA	—	—
Current OCD	—	2.4 (1)	0 (0)	NA	—	—
Current PTSD	—	0 (0)	2.8 (1)	NA	—	—
Current GAD	—	21.6 (9)	16.8 (6)	0.34	0.38	—
Past alcohol abuse or dependence	—	12 (5)	8.4 (3)	NA	—	—
Past substance abuse or dependence	—	9.6 (4)	14 (5)	NA	—	—
Eating disorders	—	12 (5)	16.8 (6)	0.31	0.4	—
On antidepressant	—	21.6 (9)	5.6 (2)	NA	—	—
On anticonvulsant	—	9.6 (4)	14 (5)	NA	—	—
On antipsychotic	—	2.4 (1)	14 (5)	NA	—	—
On lithium salts	—	7.2 (3)	8.4 (3)	NA	—	—
Overall on medication	—	38.4 (16)	33,6 (12)	2.67	0.6	—

*Footnotes*: HC = Healthy Controls; PC: Patient Controls; SA: Suicide Attempters; KW: Kruskal Wallis test; MW: Mann Whitney test; NART: National Adult Reading Test; HDRS: Hamilton Depression Rating Scale; YMRS: Young Mania Rating Scale; BDI: Beck Depression Inventory; BIS: Baratt Impulsiveness Scale; STAI: Spielberger State-Trait Anxiety Inventory; STAXI: Spielberger State-Trait Anger Inventory; CTQ: Childhood Trauma Questionnaire; NTS: Need Threat Scale; RRRS: Risk and Rescue Rating Scale; SIS: Suicide Intent Scale; OCD: Obsessive Compulsive Disorder; PTSD: Post Traumatic Stress Disorder; GAD: Generalized Anxiety Disorder; NA = Not Applicable.

As expected, HC had lower levels of subclinical depressive symptoms (BDI score only), anxiety-state and -trait, and anger-state and –trait than both patient groups. They also experienced less often childhood trauma (excepted sexual abuse).

SA had a median number of suicide attempt of 2 (min-max: 1–10), and a median age at first suicide attempt of 22 (11–43) years old. SA had higher numbers of past depressive episodes than PC (3.5 (1–13) vs. 2 (1–50) respectively, p < 10–2). Number of past depressive episodes was therefore used as covariate in the subsequent neuroimaging analyses in comparisons between patient groups (i.e. PC vs. SA). While medication was equally distributed across groups, we nevertheless controlled for this variables in analyses.

Following the Cyberball game, PC and SA had higher mean scores of social distress than HC, with no difference between SA and PC (63.5 (41–96), 65 (40–96) vs. 54 (31–87) respectively, p = 0.01).

### Functional MRI

#### Within-group analyses

**Figure 1:**
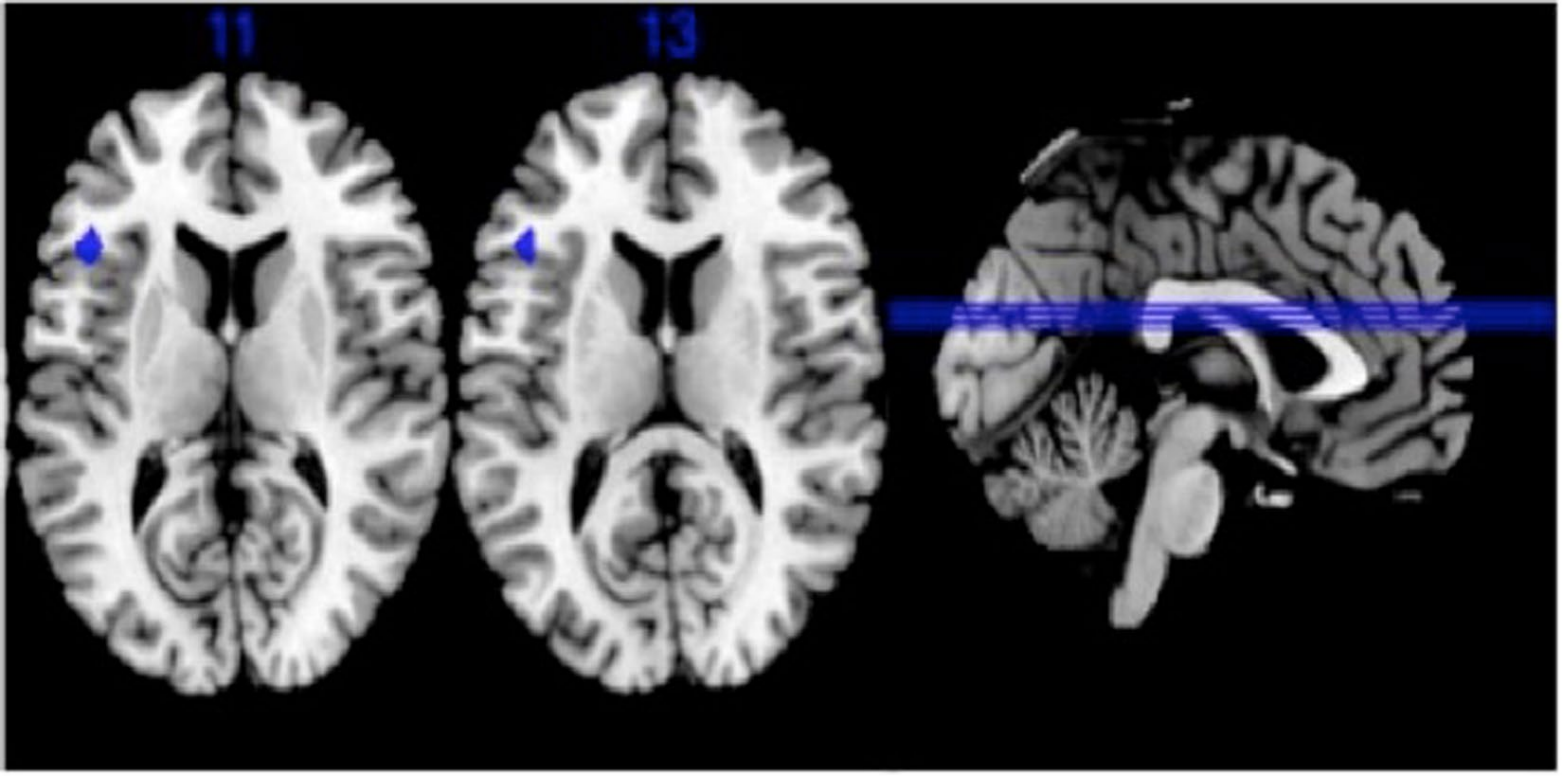
Main effect of conditions in healthy controls (voxel p-FWE corrected < 0.05, k ≥ 10) Brodmann Areas 13/45.

ANOVA analysis revealed a main effect of condition in left anterior insula/inferior frontal gyrus (Brodmann Area [BA] 13 extending to BA45, peak voxel: −43 18 7, 11 voxels, F = 14.9; Z = 5.2; voxel p-FWE = 0.002) in HC (Fig. 1), but not in PC and SA. Post hoc analyses in HC showed greater activation for both explicit social exclusion (ESE) and implicit social exclusion (ISE) vs. inclusion (INC) in anterior insula. This region has previously been associated with this contrast in healthy subjects in a recent meta-analysis^21^.

#### Between-group analyses

**Figure 2:**
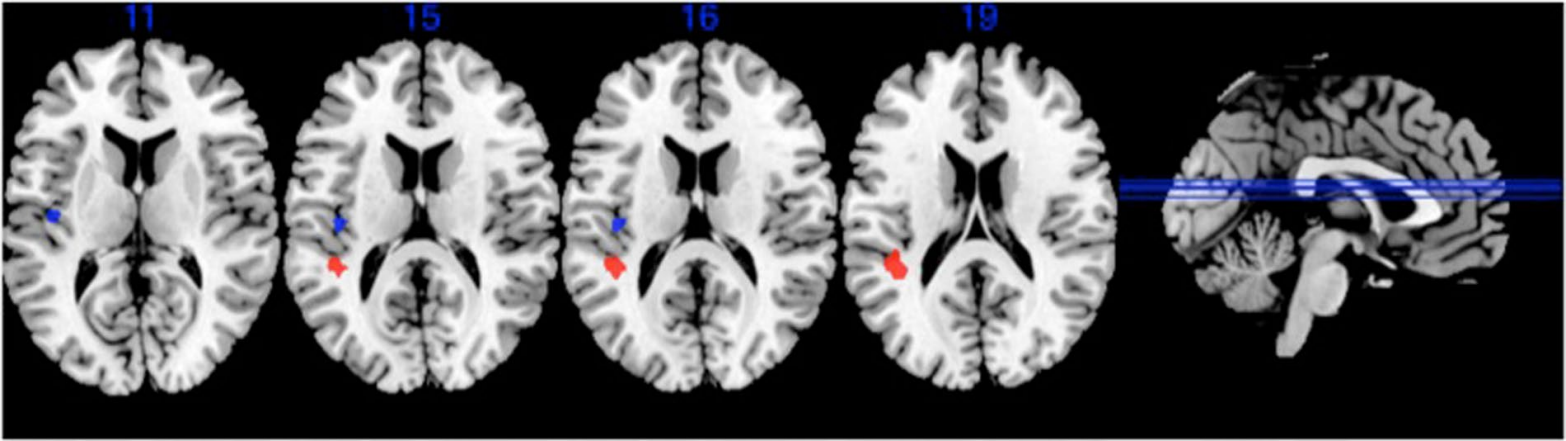
Comparison of brain activation between the three groups for the explicit exclusion (ESE) vs. inclusion (INC) conditions contrast. Significant group x condition differences in supramarginal gyrus at −39 −36 18 (red) and insula −39 −16 13 (blue) (voxel p-FWE corrected < 0.05, k ≥ 10).

ANOVA analyses revealed a group by condition interaction only for ESE vs. INC for two clusters located in left supramarginal gyrus (BA40 extending to BA13; peak voxel: −39 −36 18; 23 voxels; F = 25.2; Z = 6.28; voxel p-FEW <10^−3^) and posterior insula (BA13; peak voxel: −39 −16 13; 10 voxels; F = 18.10; Z = 5.3; voxel p-FWE = 0.001) (Fig. 2). Post-hoc analyses showed decreased activation in these regions in SA relative to both PC (after covarying for number of past depressive episodes and medication) and HC (without covariates). These results were unchanged when mood disorder type or social phobia were used as covariates.

**Figure 3:**
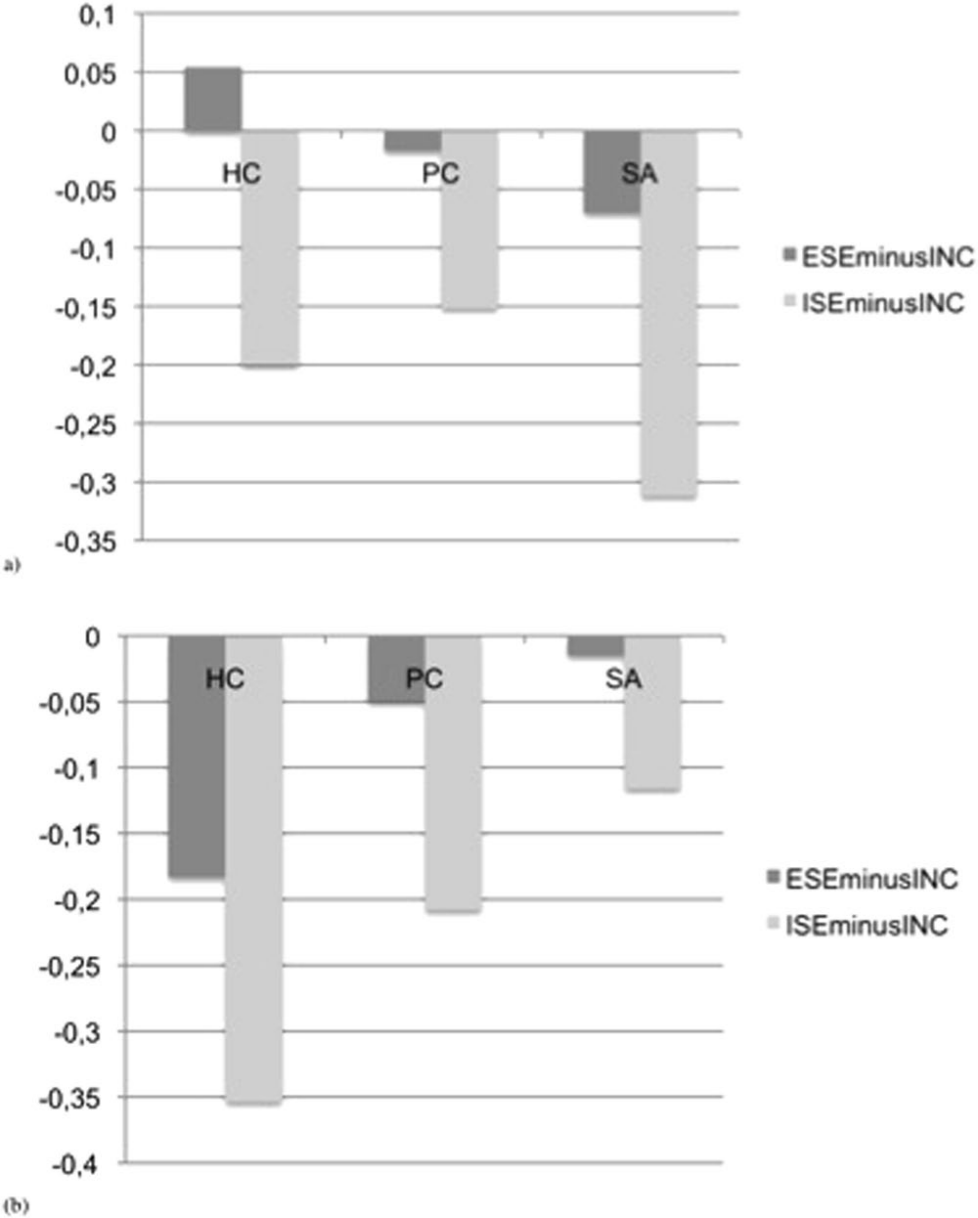
Betas values of brain activation (arbitrary units) during contrasts of interest ES vs. INC and ISE vs. INC in the three groups in (**a**) left supramarginal gyrus (−39 −36 18) and (**b**) left posterior insula (−39 −16 13). Footnotes: SA: Suicide attempters; PC: Psychiatric controls; HC: Healthy controls; ESE: explicit social exclusion; ISE: implicit social exclusion; INC: inclusion.

For explanatory purposes, Fig. 3 presents extracted beta-values for the two contrasts in the three groups. Graphs show different patterns of responses in the two clusters. In left BA40, SA showed a larger deactivation during exclusion (whether implicit or explicit), while in left posterior insula, there was a diminished deactivation during exclusion in SA.

There were no significant correlations between brain activations for ESE vs. INC at the two clusters and clinical measures of level of childhood maltreatment, anxiety, anger, impulsivity and social distress measures. There were no significant correlations between decreased cerebral activations for ESE vs. INC and number of previous suicide attempts as well as intent and lethality of the most recent and most severe suicide attempts.

## Discussion

This first study exploring the neural basis of the experience of social exclusion in SA showed two main results. Using the self-administered scale (NTS), both patient groups, although euthymic, experienced more distress than HC during the Cyberball Game, with no difference between patient groups. However, functional neuroimaging was able to discriminate patients with vs. without a history of suicidal behavior. Indeed, during the exclusion phase of the Cyberball Game, patients with a history of suicidal acts showed a different activation in left posterior insula and supramarginal gyrus (two interconnected regions^23^) compared with both psychiatric and healthy controls. First, comparing the three groups we found a reduced activity of the posterior insula in SA vs. both groups of controls during exclusion. More precisely, the posterior insula showed a reduced deactivation in SA during ESE vs. INC. While not significant, this seems to be also the case during ISE vs. INC suggesting a general alteration in brain processing of exclusion. Previous studies have reported structural, metabolic abnormalities and modified connectivity of the insula in suicidal vulnerability^2, 3, 24^. The posterior part of insula has been involved in interoceptive information^25^ and pain^26^. In borderline personality disorder patients, enhanced physical pain tolerance has been associated with decreased activation of insula during pain processing in comparison to controls^27^. Thus reduced activity of the posterior insula in our sample may be a correlate of higher tolerance to pain via repeated exposure—and attendant habituation—to painful and provocative experiences in subjects vulnerable for suicide, as suggested by the interpersonal theory of suicide^28^. Indeed, insula is part of the network underlying the acquired capability for suicide^29^, which would facilitate lethal suicidal act when occurring simultaneously with thwarted belongingness (i.e., loneliness and lack of reciprocity) and perceived burdensomeness (i.e., feeling like a liability on others). Interestingly, in a prospective 2-year observational PET study, Oquendo *et al.* ^30^ showed that greater 5HT_1A_ receptor binding potential in insula predicted more lethal attempts in depressed subjects^30^.

Second, we also found a different activity in the supramaginal gyrus in SA vs. both groups of controls during exclusion, with larger deactivation during ESE vs. INC. Reduced volumes of bilateral inferior parietal lobe have previously been reported in SA^31, 32^. Additionally, using magnetization transfer imaging, Chen *et al.* ^33^ reported impaired macromolecular structural integrity in left inferior parietal lobe in SA relative to non-attempters and HC^33^. Inferior parietal lobe is known to be involved in social cognition^34^, but also in first/third person perspective-taking^35^. Interestingly, in our study, this region did not show the same pattern of activation during ESE and ISE condition (vs. INC) in HC: left supramarginal gyrus was activated during ESE (when participants are excluded by the others) but deactivated during ISE (when particpants are told they will not participate for technical reasons) in HC. In SA, it was deactivated in both conditions suggesting that SA have difficulties taking into account the context in exclusion conditions.

Third, we have not found significant differences of activation in orbitofrontal cortex in SA as hypothesized. It may suggest that increased lateral orbitofrontal activation in SA when viewing angry faces^13, 36^ may reflect a hypersensitivity to reprobation or conflict more than to social rejection *per se*. Overall, current and previous findings support a significant and sustained sensitivity to social stressors in individuals at-risk of suicide.

Our study has several limitations. First, most of our patients were taking medications during this study. This may have influenced final findings. However, the distribution of different classes of drugs was similar between the two patient groups, which have been taken into account in analyses between patients. Moreover, patients presented with various comorbidities. In the present study, we chose not to exclude medicated patients (except patients with benzodiazepines) or patients with several comorbidities in order to maintain a reasonable level of representativeness. Importantly, when controlling for medication, mood disorder type or social phobia, our main results remained unchanged. Second, the naturalistic validity of the game is questionable as is the sensitivity of the questionnaire to measure distress at the end of the scanning session. Alternative investigations will have to be conducted. However, even if Cyberball game is not reflecting what individuals experience in their daily social interactions, Eisenberger *et al.* ^37^ have shown strong relationships between neural activity during this experimental task and real-world feelings of rejection. Moreover, individuals who are the most sensitive to experimental social rejection in the scanner are also the most sensitive to these types of experiences in their everyday lives. Third, only female participants were recruited here, enabling to exclude a gender effect and give sufficient statistical power. A similar study deserves to be conducted in men because behavioural and biological differences have been reported during Cyberball game according to gender as well as evidence for gender paradox in suicidal process. Fourth, Cyberball non-randomized block design may involve a risk of failure of non-sphericity in the low frequency range by using parametric instead non-parametric thresholding. But we decided to perform parametric analyses as always done in previous studies using this paradigm.

The main strengths of our study are the large sample size and assessment of euthymic patients to capture traits of suicidal vulnerability. The Cyberball game had never been used in subjects having a history of suicide attempt. Studies exploring influence of social stressors in vulnerable subjects are very relevant in suicidal process. Our results suggest a role for the left posterior insula and parietal regions in sensitivity to social exclusion as part of the suicidal vulnerability in women, and raise the question of the role of social perception and physical pain in the suicidal process.

## Materials and Methods

### Participants

Three groups of euthymic female participants were recruited: (1) suicide attempters (SA)—individuals with a past history of both major depressive episode and suicidal behaviour; (2) patient controls (PC)—individuals with a past history of major depressive episode but no personal history of suicidal acts; (3) healthy controls (HC)—individuals with no past history of any major DSM-IV axis I diagnosis.

Healthy controls were recruited through advertisement and among a list of volunteers from the Montpellier Academic hospital database. Patients were recruited among outpatients of the Department of Emergency Psychiatry & Post Acute Care from the Montpellier Academic hospital. Participants were first screened for inclusion criteria and then in person by a psychiatrist. All participants were Caucasian right-handed (as assessed by the Edinburgh scale^38^) females. Only females were included in this study to avoid any gender effects that may blur the results, insure a sufficient statistical power, and because most previous studies using the Cyberball game were conducted with females.

Suicidal behaviour was defined as any act carried out with some intent to die. Diagnoses were made according to DSM-IV criteria using the Mini-International Neuropsychiatric Interview, version 5.0.0. All participants had to be euthymic at the time of scanning, as indicated by a Hamilton Depression Rating Scale score < 7 and a Young Mania Rating Scale score < 7. Other exclusion criteria were a lifetime history of severe head trauma, CNS disorder, schizophrenia, and a history of alcohol or drug abuse or dependence within the past 12 months.

Local Ethics Committee (CPP Sud Mediterranée IV, CHU Montpellier) approved the study protocol. All experimental methods were carried out in accordance with the ethical guidelines determined by the National Ministry of Health, Labour and Welfare and the Declaration of Helsinki. All participants provided written informed consent before entering the study. Subjects received 100€ for their participation in the study.

In total, 120 participants were examined. Two HC were unable to remain in the scanner because of anxiety. For technical reasons, data from two PC were not available. Moreover, we excluded from analyses patients taking benzodiazepines. In sum, data from 36 SA, 41 PC, and 28 HC were analyzed.

### Clinical assessment

We administered the French version of the National Adult Reading Test (NART)^39^ to provide an estimate of verbal IQ; the Beck Depression Inventory (BDI) for a subjective measure of depressive state; the Spielberger Anxiety Scale (STAI)—state^40^ for current level of anxiety; and the State-Trait Anger Expression Inventory (STAXI)-state^41^ for current level of anger. The lethality and the intent of the last and the most severe (according reported medical consequences) suicidal acts were assessed with the Risk Rescue Rating Scale (RRRS) and the Suicide Intent Scale (SIS)^42^.

We also assessed various personality traits including impulsivity with the Barratt Impulsiveness Scale (BIS-10)^43^, trait anger with the STAXI-trait^41^, and trait anxiety with the STAI-trait^40^. Finally, we measured a history of childhood maltreatment with the Childhood Trauma Questionnaire (CTQ)^44^.

### Cyberball Game

Functional Magnetic Resonance Imaging (fMRI) scans were acquired while participants played the Cyberball game, a virtual ball-tossing game^20^. The Cyberball game is a validated paradigm to study social exclusion and has been widely used in fMRI studies. Participants were instructed that they would play with two other players, also in fMRI scanners. In reality, participants were playing with a preset computer program and were given a cover story to ensure that they believed the other players were real. The Cyberball game comprises three successive conditions. In the first condition (Implicit Social Exclusion, ISE), the participant watched the other “players” play the Cyberball game. Participants were told that, because of technical difficulties, the link to the other two scanners could not yet be made and thus, at first, they would only watch but not play with the other two players. This cover story was intended to allow participants to view a scene visually identical to the exclusion condition (Explicit Social Exclusion, ESE) without participants experiencing exclusion by the other participants. In the second condition (inclusion, INC), participants played with the other two players and received the ball as many times as virtual players. In the final condition (ESE), participants were progressively excluded with the two other players not throwing the ball to the participant anymore. Each run consisted of 60 throws by condition, i.e. 180 throws in total for the whole session. The computer players waiting 0.5–3.0 seconds before making a throw to heighten the sense that the participant was actually playing with other individuals. ESE included 8 throws to the participant during an initial transition phase toward total exclusion.

Following completion of the Cyberball task, participants completed the Need-Threat Scale (NTS)^20^ to measure social distress associated with being excluded during the game. The NTS assesses 12 subjectively experienced consequences of being excluded during the game, including ratings of self-esteem (“I felt liked”), belongingness (“I felt rejected”), meaningfulness (“I felt invisible”), and control (“I felt powerful”), on a scale ranging from 1 = “not at all” to 5 = “very much”. Items were reverse-coded when appropriate and averaged to create a composite score.

### Image Acquisition

Imaging acquisition was done in the Neuroradiology Department - I2FH (Academic Hospital of Montpellier) - using a 1.5T whole-body MRI system (MAGNETON AVANTO, Siemens, Erlangen, Germany) equipped with a standard 12-channel receive-only head coil. Sixty volumes of BOLD echo planar images (EPI) were obtained during the Cyberball Game. Gradient-Echo EPI images characteristics were as follows: TR = 2 sec, TE = 40 ms, FOV = 220 mm, 25 axial slices (5 mm slice thickness), slice gap = 0.5 mm, voxel size = 3.43 × 3.43 × 5 mm, flip angle 90°. The slices were covering a region extending from the vertex to lower parts of the cerebellum.

A 3D magnetization-prepared, rapid acquisition gradient echo (MP-RAGE) sequence was also obtained for each participant with the following parameters: TR = 2100 ms, TE = 4.1 ms, IR = 1100 ms, 15° flip angle, PAT = 2, aligned with the corpus callosum, voxel-size 0.98 × 0.98 × 1 mm, 160 transversal slices.

### fMRI Data Analysis

Data were analyzed using SPM12 (Wellcome Department of Imaging Neuroscience, London, UK) implemented in Matlab R2015 (Mathworks, Inc., Natick, MA) using a block-designed model. The 5 first volumes of each fMRI run were discarded due to the time of launch of the Cyberball task synchronized with fMRI acquisition. The following 55 volumes were retained for functional analysis for each condition (ISE, INC and ESE). In total 165 volumes were analyzed. EG-EPI data were re-oriented to the anterior commissure, slice-time corrected, realigned to the first volume, co-registered, normalized to T1 template (provided by Montreal Neurological Institute MNI), and smoothed with an 8-mm FWHM Gaussian filter.

Contrast images were estimated for ESE vs. INC, ISE vs. INC and ESE vs. ISE conditions for every participant using a first-level general linear model. Realignment parameters have been added in the regressor to remove specific activation of head movement of the subject and 128 seconds high-pass filter was used to remove non-physiological slow signal shifts. To check the validity of our experiment, we first conducted a one-way ANOVA at the whole brain level for the main effect of condition in HC. Significance threshold was set at voxel level p < 0.05, family-wise error (FWE) corrected for multiple comparisons with k ≥ 10 voxels. Then, we conducted a twoway mixedmodel ANOVA, at the whole-brain level for the interaction between Group and Condition. Significance threshold was set at voxelwise FWE-corrected p < 0.05, with k ≥ 10 voxels. Post hoc tests were performed using two sample T-tests with an inclusive mask of regions showing a significant group by condition interaction in the ANOVA (voxel p-uncorrected p < 0.001, k ≥ 10 voxels). The website http://sprout022.sprout.yale.edu/mni2tal/mni2tal.html and xjView toolbox were used for anatomical localization. Coordinates are reported in Talairach space. Beta values were then extracted to measure correlations with social distress scores and clinical variables.

### Statistical Analyses

Clinical and behavioral analyses were carried out with SPSS Statistics 23 (SPSS, Inc., Chicago). Clinical and behavioural quantitative data were compared between the three groups with Kruskal-Wallis tests and between pairs of groups with Mann-Whitney U tests. Associations between qualitative variables and groups were calculated with chi-square tests. All correlation analyses of brain activation with clinical measures were conducted using Spearman test. The alpha level was set *a priori* at 0.05.
